# Sex differences in autonomic function following maximal exercise

**DOI:** 10.1186/s13293-015-0046-6

**Published:** 2015-12-01

**Authors:** Rebecca M. Kappus, Sushant M. Ranadive, Huimin Yan, Abbi D. Lane-Cordova, Marc D. Cook, Peng Sun, I. Shevon Harvey, Kenneth R. Wilund, Jeffrey A. Woods, Bo Fernhall

**Affiliations:** Department of Health and Exercise Science, Appalachian State University, 111 Rivers Street, 038 HCC, Boone, NC 28608-2071 USA; Department of Kinesiology, Nutrition, and Rehabilitation, University of Illinois at Chicago, Chicago, IL USA; Department of Anesthesiology, Mayo Clinic, Rochester, MN USA; Department of Kinesiology, East Carolina, Greensboro, NC USA; Department Health and Human Physiology, University of Iowa, Iowa City, IA USA; Key Laboratory of Adolescent Health Assessment and Exercise Intervention, Ministry of Education, East China Normal University, Shanghai, China; Department of Health and Kinesiology, Texas A & M University, College Station, TX USA; Department of Kinesiology and Community Health, University of Illinois at Urbana-Champaign, Urbana, IL USA

**Keywords:** Heart rate variability, Heart rate recovery, Blood pressure variability, Sympathetic activity, Vagal modulation

## Abstract

**Background:**

Heart rate variability (HRV), blood pressure variability, (BPV) and heart rate recovery (HRR) are measures that provide insight regarding autonomic function. Maximal exercise can affect autonomic function, and it is unknown if there are sex differences in autonomic recovery following exercise. Therefore, the purpose of this study was to determine sex differences in several measures of autonomic function and the response following maximal exercise.

**Methods:**

Seventy-one (31 males and 40 females) healthy, nonsmoking, sedentary normotensive subjects between the ages of 18 and 35 underwent measurements of HRV and BPV at rest and following a maximal exercise bout. HRR was measured at minute one and two following maximal exercise.

**Results:**

Males have significantly greater HRR following maximal exercise at both minute one and two; however, the significance between sexes was eliminated when controlling for VO_2_ peak. Males had significantly higher resting BPV-low-frequency (LF) values compared to females and did not significantly change following exercise, whereas females had significantly increased BPV-LF values following acute maximal exercise. Although males and females exhibited a significant decrease in both HRV-LF and HRV-high frequency (HF) with exercise, females had significantly higher HRV-HF values following exercise. Males had a significantly higher HRV-LF/HF ratio at rest; however, both males and females significantly increased their HRV-LF/HF ratio following exercise.

**Conclusions:**

Pre-menopausal females exhibit a cardioprotective autonomic profile compared to age-matched males due to lower resting sympathetic activity and faster vagal reactivation following maximal exercise. Acute maximal exercise is a sufficient autonomic stressor to demonstrate sex differences in the critical post-exercise recovery period.

## Background

Measures of autonomic nervous system function such as heart rate variability (HRV), blood pressure variability (BPV), and heart rate recovery (HRR) provide important information regarding autonomic regulation [1]. Low heart rate variability (HRV) is considered prognostic for future risk of cardiovascular disease (CVD) and has been linked to conditions such as coronary artery disease, hypertension, and heart failure [2, 3]. Elevated BPV is associated with essential hypertension [4] and increased cardiovascular risk, independent of average blood pressure values [5, 6]. Patients with high BPV have greater end-organ damage and left ventricular mass index than hypertensive patients with the same mean 24-h blood pressure values with a lower BPV [7]. Further insight into autonomic function can be gained following exercise because of the significant effect exercise can have on autonomic modulation [8, 9]. A delayed heart rate recovery (HRR) during the first minute following graded exercise is a predictor of overall mortality in individuals with and without cardiovascular disease, independent of workload, the presence or absence of myocardial perfusion defects, and changes in heart rate during exercise [10].

Females display lower sympathetic activity and increased cardiac vagal modulation which could reflect the lower prevalence of arrhythmias blood pressure compared to males [11]. However, this may not be maintained following recovery after a short supramaximal exercise bout [12]. Maximal exercise can affect the autonomic nervous system by increasing sympathetic activity and decreasing vagal modulation [13], and there is a delay in the return to parasympathetic dominance following submaximal exercise [14]. However, little is known with regard to sex differences at rest and following maximal exercise in several established measures of autonomic function. It is unknown if an acute maximal bout of exercise will affect young, untrained men and women differently and if autonomic recovery is depressed in both males and females. We hypothesize that females will have enhanced parasympathetic dominance at rest compared to males, but an acute maximal exercise bout will lead to elevated sympathetic dominance in both males and females coupled with delayed parasympathetic reactivation in females.

## Methods

### Participants

Seventy-one (31 males and 40 females) healthy, nonsmoking, normotensive subjects between the ages of 18 and 35 participated in this study. All subjects were recruited from a local university and community population through flyers or word of mouth. Subjects were classified as sedentary based on their exercise habits for the past 6 months (no structured exercise activity of any kind lasting longer than 30 min more than one time per week). Participants completed a health history questionnaire and were free of any cardiovascular disease, hypertension, or hyperlipidemia. None of the subjects were taking any medications except oral contraceptives. The study followed the procedures for protection of human participants as provided in the 1975 Declaration of Helsinki. Prior to any data collection, all participants signed informed consent, and the study was approved by the University of Illinois at Urbana-Champaign institutional review board.

### Study design

**Fig. 1:**
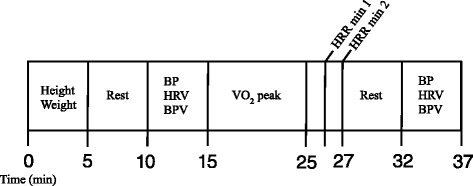
Schematic outline of study protocol. *Min* minutes, *BP* blood pressure, *HRV* heart rate variability, *BPV* blood pressure variability, *HRR* heart rate recovery

Subjects reported to the lab for one visit. All women were tested in the early follicular phase or during the placebo phase of oral contraceptives. Subjects were instructed to be at least 4 h postprandial and to abstain from caffeine and alcohol for at least 12 h and abstain from exercise for at least 24 h before testing. Measurements of height were taken using a stadiometer to the nearest 0.5 cm, and body weight was obtained using a beam balance platform scale to the nearest 0.5 kg. Subjects then rested quietly in a supine position for 5 min before systolic and diastolic blood pressure (BP) measurements were taken using an automated oscillometric cuff (HEM-907 XL; Omron, Japan). Brachial BP measurements were repeated, and if values were within 5 mm Hg of each other, the average of the two values was used for analysis. If measurements were not within 5 mm Hg, readings were taken until two values within 5 mm Hg were obtained. American Heart Association guidelines were followed except all measurements were performed in the supine position to provide a more stable and reproducible blood pressure. HRV and BPV measurements were assessed using finger plethysmography (Finometer, FMS, the Netherlands; see further description below). Subjects were supine in a quiet, darkened room and underwent 5 min of paced breathing to a metronome resulting in 12 breaths per minute. Following these measures, the participants underwent a VO_2_ peak test. The VO_2_ peak test was done on an upright, stationary cycle ergometer (Lode Excaliber Sport, Groningen, Netherlands). Heart rate (HR) was recorded for the 2 min following cessation of maximal exercise to determine HRR. Minute 1 was an active recovery with the subject easily pedaling with no resistance. Minute 2 was an inactive recovery, with the subject sitting quietly on the ergometer. Following the 2-min recovery period, subjects were then allowed to rest in a comfortable, supine position in a quiet, dark room. Approximately 5 min were used to setup and calibrate the plethysmograph and subjects then underwent 5 min of BPV and HRV measurements with paced breathing (Fig. 1).

### Blood pressure variability

Beat-to-beat blood pressures were measured using finger plethysmography (FMS, Amsterdam, The Netherlands). This method yields valid assessment of blood pressure for the changes that occur from heart beat to heart beat and includes both short- and long-lasting changes [15]. Blood pressure spectral peaks were detected using an established algorithm, and Fast Fourier transform algorithms were used to convert the data into frequency spectra.

### Heart rate variability

R-R intervals were derived from beat-to-beat blood pressure pulse intervals using finger plethysmography (FMS, Amsterdam, The Netherlands). Finger plethysmography-derived peak-to-peak intervals are highly correlated with ECG R-R intervals, with similar variability, a high degree of agreement (determined by Bland-Altman analysis); there are no significant differences between the HRV obtained from the two different methods [16]. The upstroke is determined using the pressure signal with a resolution of 2 ms, and the interval between the two consecutive upstrokes is measured.

### BPV and HRV analysis

Both BPV and HRV data were analyzed in the frequency domain (WinCPRS, Absolute Aliens Oy, Turku, Finland). Data were visually inspected, and any artifact was eliminated prior to analysis. In the frequency domain, the two primary components are low-frequency (LF; 0.04–0.15 Hz) and high-frequency (HF; 0.15–0.40 Hz) spectra. HRV measures provide information primarily on vagal modulation with the LF power spectrum reflecting both sympathetic and parasympathetic modulation and HF acting as a surrogate marker of parasympathetic modulation of the R-R intervals [12]. The LF/HF ratio is used as an indicator of sympathovagal dominance [17]. BPV is thought to provide insight on sympathetic modulation through the measure of LF power [18]. All data acquisition and post-acquisition analyses were carried out in accordance with the Task Force of the European Society of Cardiology and North American Society of Pacing and Electrophysiology [19].

### VO_2_ peak test

Following a warm-up of 1 min of unloaded cycling, subjects began pedaling at 50 watts (W). The resistance was then increased 30 W every 2 min until test termination. HR was measured using a heart rate monitor (Polar Electro, Woodbury, NY) and expired air was collected and analyzed using a Quark b2 breath-by-breath metabolic system (Cosmed, Rome, Italy). The sampling interval used to determine VO_2_ peak was the highest 20 s average VO_2_. All participants reached volitional fatigue or an inability to maintain pedal rate above 60 rpm, upon which the test was terminated. Following the VO_2_ peak test, test results were examined and all participants were shown to have satisfied two or more of the following four criteria: (1) a respiratory ratio of 1.1 or greater; (2) a plateau in HR despite an increase in workload; (3) a final rate of perceived exertion (RPE) score of 17 or greater on the Borg scale (scale 6–20); and/or d) an increase in work rate that elicited an increase in VO_2_ of ≤150 mL/min, indicative of a plateau in oxygen consumption [20, 21].

### Statistical analyses

Pre- and post-maximal exercise test values were analyzed between sexes using a 2 × 2 repeated measures analysis of variance (ANOVA) using SPSS 19.0 (Armonk, NY: IBM Corp.). When the interaction was significant (*p* < 0.05), the responses were further evaluated using paired samples *t* tests within each group and independent *t* tests between sexes. Data were tested for normality using Shapiro-Wilks, and all HRV and BPV variables were not normally distributed, so data were log transformed before statistical analyses. Log transformed data are reported in figures.

## Results

**Fig. 2:**
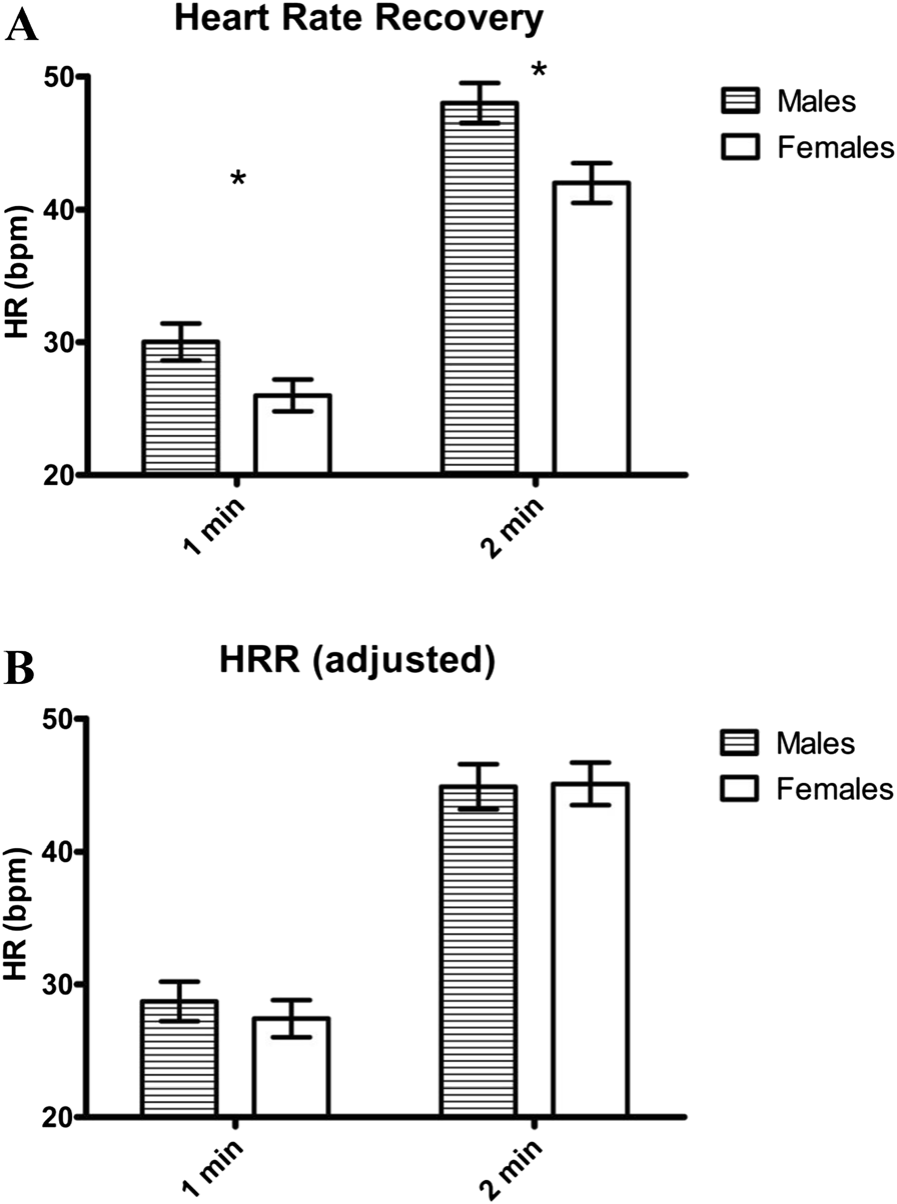
Heart rate recovery following maximal exercise. **a** Heart rate recovery, raw data. **b** Heart rate recovery after adjusting for VO_2_ peak. *HRR* heart rate recovery, *HR* heart rate, *bpm* beats per minute, *1-min* heart rate recovery in the first minute after exercise, *2 min* heart rate recovery in the second minute after exercise. *p < 0.05 sex differences

Subject descriptives are reported in Table 1. There were significant sex differences in height and VO_2_ peak. Age by sex frequencies are shown in Table 2. HRR (raw data) are shown in Fig. 2a and demonstrates that males, compared to females, have significantly greater HRR following maximal exercise at both minute one and two (*p* < 0.05). After controlling for VO_2_ peak, the significance between sexes was eliminated and adjusted means are shown in Fig. 2b.

**Table 1:** Descriptive Statistics

	Males (*n* = 31)	Females (*n* = 40)	Total (*n* = 71)
Age (years)	24 ± 1	25 ± 1	24 ± 0.5
Height (cm) *	177.9 ± 1.0	163.4 ± 1.1	170.2 ± 1.2
Weight (kg)	82.7 ± 2.4	73.0 ± 4.1	79.2 ± 2.6
BMI (kg/m^2^)	26.1 ± 0.8	27.1 ± 1.3	27.1 ± 0.8
VO_2_peak (ml/kg/min) *	39.2 ± 1.3	28.8 ± 1.0	33.3 ± 1.0
HR rest (bpm) *	62 ± 1	67 ± 2	65 ± 1
HR peak (bpm)	184 ± 2	179 ± 2	180 ± 1
HR at 1 min recovery (bpm)	153 ± 2	153 ± 2	153 ± 2
HR at 2 min recovery (bpm)	136 ± 2	136 ± 2	136 ± 1

All data presented as mean ± standard error. *p < 0.05 between sexes

Raw data for HRV and BPV are reported in Table 3. Data were not normally distributed; therefore, only log transformed data are presented in figures.

**Table 2:** Age by sex frequencies

Age	Male		Females	
	Frequency	Percent	Frequency	Percent
19	1	3.2	1	2.5
20	7	22.6	2	5
21	5	16.1	8	20
22	3	9.7	9	22.5
23	3	9.7	1	2.5
24	1	3.2	2	5
25	2	6.5	1	2.5
26	1	3.2	3	7.5
27	2	6.5	2	5
28	1	3.2	2	5
29	0	0	1	2.5
30	0	0	3	7.5
31	2	6.5	1	2.5
32	0	0	1	2.5
33	1	3.2	2	5
34	0	0	1	2.5
35	2	6.5	0	0
TOTAL	31	100	40	100

**Table 3:** Raw data

	Males		Females	
	Pre	Post	Pre	Post
BPV-LF (mmHg^2^)	10.16 ± 1.90	8.02 ± 1.40	5.31 ± 0.71	8.22 ± 1.33
BPV-HF (mmHg^2^)	4.21 ± 0.94	2.11 ± 0.33	4.05 ± 0.54	2.19 ± 0.24
BPV-LF/HF (%)	351.05 ± 58.69	490.42 ± 79.21	219.33 ± 55.23	473.21 ± 74.55
HRV-LF (ms^2^)	2096.48 ± 505.31	996.67 ± 872.63	1415.31 ± 418.52	1439.80 ± 833.43
HRV-HF (ms^2^)	3348.67 ± 764.54	1921.74 ± 1789.78	4236.40 ± 791.31	1732.54 ± 1234.04
HRV-LF/HF (%)	135.31 ± 25.39	182.74 ± 35.38	51.79 ± 23.89	158.47 ± 33.30

All data presented mean ± SEM

**Fig. 3:**
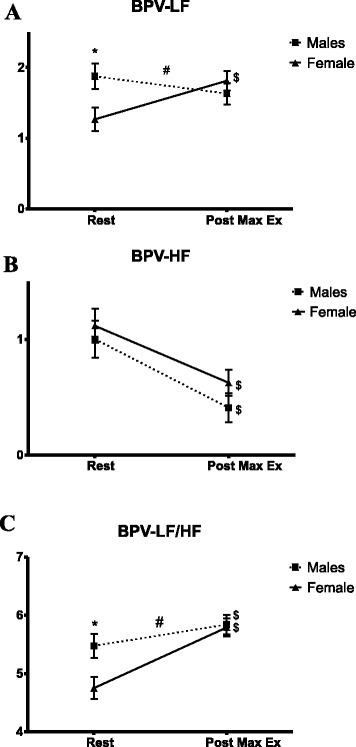
Blood pressure variability before and following maximal exercise. **a** Blood pressure variability, low frequency. **b** Blood pressure variability, high frequency. **c** Blood pressure variability low-frequency/high-frequency ratio. *BPV* blood pressure variability, *LF* low frequency, *HF* high frequency. * *p* < 0.05 sex differences; # *p* < 0.05 interaction (time × sex); $ *p* < 0.05 time effect

BPV data are displayed in Fig. 3. Although males had significantly higher LF values compared to females at rest and did not significantly change following exercise, females had significantly increased LF values following acute maximal exercise, eliminating sex differences (Fig. 3a). HF decreased significantly and similarly in both males and females (Fig. 3b). LF/HF increased significantly in both males and females, with greater increase in females, eliminating the resting differences between sexes (Fig. 3c).

**Fig. 4:**
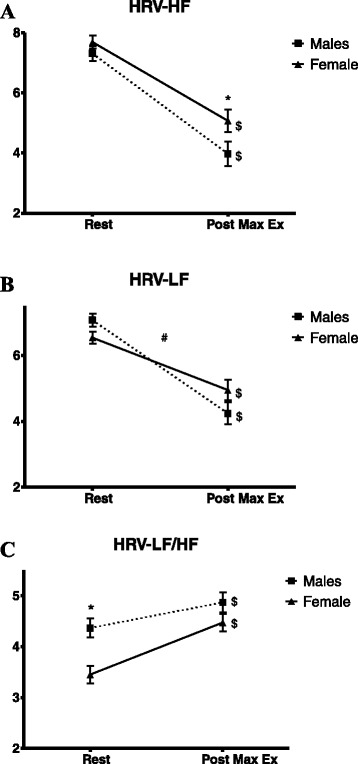
Heart rate variability before and following maximal exercise. **a** Heart rate variability, low frequency. **b** Heart rate variability, high frequency. **c** Heart rate variability low-frequency/high-frequency ratio. *HRV* heart rate variability, *LF* low frequency, *HF* high frequency. * *p* < 0.05 sex differences; # *p* < 0.05 interaction (time × sex); $ *p* < 0.05 time effect

HRV data are displayed in Fig. 4. Both sexes exhibited a significant decrease in both LF (Fig. 4a) and HF (Fig. 4b), although females had significantly higher HF values following exercise. Both males and females significantly increased their LF/HF ratio following exercise (Fig. 4c); however, males had a significantly higher LF/HF ratio at rest and exercise eliminated these sex differences. When using VO_2_ peak as a covariate when analyzing BPV and HRV data, any initial significance (*p* < 0.05) remained, and results were not affected.

## Discussion

The major findings of this study are as follows: (1) males had greater HRR than females, and differences were eliminated after controlling for VO_2_ peak; (2) females had significantly lower BPV-LF and BPV-LF/HF values at rest compared to males but had more pronounced increases with maximal exercise; (3) males displayed a higher HRV-LF/HF ratio at rest, and females had higher HRV-HF values following exercise. Overall, this suggests greater vagal modulation and potentially greater vagal reactivation following maximal exercise in females while males display elevated resting sympathetic dominance.

Resting measures of autonomic function are predictive of cardiovascular outcome and abnormal autonomic recovery following exercise is associated with potentially fatal arrhythmias, sudden cardiac death, and ischemic heart disease [22]. Females have a lower incidence of arrhythmias and sudden cardiac death compared to males [23], suggesting enhanced autonomic function. These sex differences could be due to the potential cardioprotective effect of ovarian hormones [24], as estrogen improves vasomotor tone and vascular integrity, lowers blood pressure, and improves lipid profiles and cholesterol metabolism [25]. In addition, ovarian hormones have also been shown to influence autonomic cardiovascular regulation [26] by preventing sympathovagal imbalance [27] and improving baroreflex sensitivity [28]. The females in this study were pre-menopausal and therefore can be assumed to have elevated estrogen levels compared to the male cohort; however, we did not directly measure ovarian hormones.

Further insight into sex differences may be discerned by studying responses during the recovery period following maximal exercise, when the autonomic nervous system is transitioning from sympathetic to parasympathetic dominance. During exercise, rapid parasympathetic withdrawal and gradual increased sympathetic activity and circulating catecholamines contribute to the increase in HR [29]. HR decreases following maximal exercise reflect both catecholamine clearance (we did not measure circulating catecholamine levels nor catecholamine clearance rates in this study) and parasympathetic reactivation and is predictive of all-cause mortality and sudden death [10]. Although HRR was significantly different between sexes, after controlling for VO_2_ peak, there were no differences between males and females at either minute one or minute two following maximal exercise. This finding supports previous findings demonstrating a direct relationship between HRR and aerobic fitness levels [30] and suggests that sex does not impact HRR following maximal exercise in young, healthy individuals, but instead, HRR is dependent on cardiovascular fitness. Similar findings have also been shown in an older (mean age = 56 years) clinical population following exercise treadmill testing [31]. Prior studies have also shown that there are no sex differences between HRR and all-cause mortality [32, 33]. In addition, despite low HRR (10–15 bpm) being linked with increased mortality, values above 15–20 bpm are not associated with further improvements in prognosis [10]. Therefore, in this particular population with HRR values well above 20 bpm, this measure alone may not be adequate for estimating cardiovascular risk in young, healthy individuals.

HRV-LF values between males and females were similar at rest, and both sexes decreased HRV-LF significantly following exercise, consistent with previous research [34]. However, males had a significantly greater decrease in LF following maximal exercise. Since the LF power spectrum represents both sympathetic and parasympathetic activity, it is not possible to discern which arm of the autonomic nervous system is responsible for this sex difference. However, our HRV data may suggest that the autonomic system in males is more sensitive to maximal exercise, with a more dramatic fluctuation in HRV-LF.

Although there were no sex differences at rest in HRV-HF and both males and females decreased their HF values following maximal exercise, females displayed higher HF values following exercise, suggesting greater vagal modulation and potentially greater vagal reactivation following maximal exercise in females. Data from the Framingham study showed sex differences in the incidence of adverse events related to autonomic dysfunction, such as sudden cardiac death and arrhythmias. Despite an increase in the incidence of sudden cardiac death with age in both males and females, at all ages combined, women experience approximately half of the annual rate of sudden cardiac death than men [35]. In addition, men develop atrial fibrillation at one and a half times the rate of women even after adjusting for age and other cardiovascular diseases [36]. Although our study showed no sex differences in HRV-HF at rest, it should be noted that the female cohort demonstrated enhanced sympathovagal balance at rest and furthermore, our HRV data suggests that this cardioprotection in females is present in the post-exercise period, which is a critical time for autonomic recovery. Interestingly, HRV responses following exercise occurred independent of fitness (VO_2_ peak), which was not the case for HRR. These discrepancies could be due to the differences in these two measures; HRV is thought to reflect phasic changes in vagal outflow whereas HRR reflects mean cholinergic signaling at the sinoatrial nodal junction [37].

Although there is a direct relationship between HRR and fitness levels [30], the relationship between HRV and fitness is inconsistent, with some studies demonstrating a link [38, 39] and others showing no relationship [40, 41]. These discrepancies could reflect differences in how HRV was measured, subject characteristics, age and training status. Tulppo et al. [42] showed no differences in resting HRV between groups of differing fitness levels despite significant differences in resting HR; however, the high fitness group demonstrated enhanced vagal function during exercise compared to the low-fitness group. However, HRV was measured during the actual exercise bout and not during the recovery period, as with our study. Our HRV and HRR results suggest that sex has a significant impact on HRV following maximal exercise independent of fitness levels, while HRR is more affected by fitness levels as opposed to sex differences.

The HRV-LF/HF ratio was significantly greater in males at rest, suggesting elevated sympathetic modulation/dominance, and increased in both males and females following exercise, which supports previous studies [11, 12]. However, it is important to note that the increase in the LF/HF ratio seen in the current study is most likely driven by the decrease in HRV-HF and a less sharp decline in LF in females. Interestingly, males had no significant changes in BPV-LF and even displayed a slight decrease while females significantly increased their BPV-LF values following exercise, thereby eliminating any sex differences initially seen at rest. Females demonstrated the expected response in BPV-LF values following exercise (increased sympathetic activity) whereas males had similar values in BPV-LF both before and following exercise. Thus, our findings show that females exhibit both a greater change in, but similar absolute levels of sympathetic modulation (BPV-LF), and greater parasympathetic modulation (HRV-HF) following maximal exercise.

## Limitations

We did not directly measure ovarian hormone levels. However, we did test all females during the early follicular phase of their menstrual cycle, assuring all females were relatively similar in their hormone levels. In addition, because all females were pre-menopausal, we can confirm the estrogen differences between males and females.

We did not perform direct measures of sympathetic activity. However, beat-to-beat blood pressure and finger plethysmography has been validated against invasive measures and is an accurate measure of HRV and BPV [16, 43], allowing for the noninvasive determination of autonomic nervous system activity.

## Conclusions

In conclusion, our results suggest that young females demonstrate enhanced autonomic function at rest and following an acute exercise bout. This was demonstrated in lower BPV-LF values at rest and higher HRV-HF values following exercise compared to males, suggesting greater vagal modulation and vagal reactivation following maximal exercise in females with males displaying elevated resting sympathetic dominance. We also found that an acute maximal exercise bout was a sufficient autonomic stressor to demonstrate sex differences in the critical post-exercise recovery period. Together, these results indicate that pre-menopausal females exhibit differences in autonomic function compared to age-matched males due to lower resting sympathetic activity and faster vagal reactivation following maximal exercise. Because elevated sympathetic activity, even in healthy adults, is linked to arterial remodeling [44] and fatal arrhythmias [45], these sex differences in autonomic function may contribute to the lower prevalence of cardiovascular disease and arrhythmias in females [23].

## References

[1] Levy MN (1990). Autonomic interactions in cardiac control. Ann N Y Acad Sci.

[2] La Rovere MT, Pinna GD, Maestri R, Mortara A, Capomolla S, Febo O (2003). Short-term heart rate variability strongly predicts sudden cardiac death in chronic heart failure patients. Circulation.

[3] Palatini P (1999). Heart rate as a risk factor for atherosclerosis and cardiovascular mortality: the effect of antihypertensive drugs. Drugs.

[4] Mancia G, Ferrari A, Gregorini L, Parati G, Pomidossi G, Bertinieri G (1983). Blood pressure and heart rate variabilities in normotensive and hypertensive human beings. Circ Res.

[5] Stolarz-Skrzypek K, Thijs L, Richart T, Li Y, Hansen TW, Boggia J (2010). Blood pressure variability in relation to outcome in the International Database of Ambulatory blood pressure in relation to Cardiovascular Outcome. Hypertens Res.

[6] Rothwell PM, Howard SC, Dolan E, O'Brien E, Dobson JE, Dahlof B (2010). Prognostic significance of visit-to-visit variability, maximum systolic blood pressure, and episodic hypertension. Lancet.

[7] Frattola A, Parati G, Cuspidi C, Albini F, Mancia G (1993). Prognostic value of 24-hour blood pressure variability. J Hypertens.

[8] Curtis BM, O'Keefe JH (2002). Autonomic tone as a cardiovascular risk factor: the dangers of chronic fight or flight. Mayo Clin Proc.

[9] Hautala A, Tulppo MP, Makikallio TH, Laukkanen R, Nissila S, Huikuri HV (2001). Changes in cardiac autonomic regulation after prolonged maximal exercise. Clin Physiol.

[10] Cole CR, Blackstone EH, Pashkow FJ, Snader CE, Lauer MS (1999). Heart-rate recovery immediately after exercise as a predictor of mortality. N Engl J Med.

[11] Ramaekers D, Ector H, Aubert AE, Rubens A, Van de Werf F (1998). Heart rate variability and heart rate in healthy volunteers. Is the female autonomic nervous system cardioprotective?. Eur Heart J.

[12] Mendonca GV, Heffernan KS, Rossow L, Guerra M, Pereira FD, Fernhall B (2010). Sex differences in linear and nonlinear heart rate variability during early recovery from supramaximal exercise. Appl Physiol Nutr Metab.

[13] Schatzkin A, Cupples LA, Heeren T, Morelock S, Kannel WB (1984). Sudden death in the Framingham Heart Study. Differences in incidence and risk factors by sex and coronary disease status. Am J Epidemiol.

[14] Kaikkonen P, Nummela A, Rusko H (2007). Heart rate variability dynamics during early recovery after different endurance exercises. Eur J Appl Physiol.

[15] Imholz BP, Langewouters GJ, van Montfrans GA, Parati G, van Goudoever J, Wesseling KH (1993). Feasibility of ambulatory, continuous 24-hour finger arterial pressure recording. Hypertension.

[16] Selvaraj N, Jaryal A, Santhosh J, Deepak KK, Anand S (2008). Assessment of heart rate variability derived from finger-tip photoplethysmography as compared to electrocardiography. J Med Eng Technol.

[17] Pagani M, Lombardi F, Guzzetti S, Rimoldi O, Furlan R, Pizzinelli P (1986). Power spectral analysis of heart rate and arterial pressure variabilities as a marker of sympatho-vagal interaction in man and conscious dog. Circ Res.

[18] Malliani A, Pagani M, Lombardi F, Cerutti S (1991). Cardiovascular neural regulation explored in the frequency domain. Circulation.

[19] TaskForce (1996). Heart rate variability: standards of measurement, physiological interpretation and clinical use. Task Force of the European Society of Cardiology and the North American Society of Pacing and Electrophysiology. Circulation.

[20] Taylor HL, Buskirk E, Henschel A (1955). Maximal oxygen intake as an objective measure of cardio-respiratory performance. J Appl Physiol.

[21] Edvardsen E, Scient C, Hansen BH, Holme IM, Dyrstad SM, Anderssen SA (2013). Reference values for cardiorespiratory response and fitness on the treadmill in a 20- to 85-year-old population. Chest.

[22] Billman GE (2002). Aerobic exercise conditioning: a nonpharmacological antiarrhythmic intervention. J Appl Physiol.

[23] Larsen JA, Kadish AH (1998). Effects of gender on cardiac arrhythmias. J Cardiovasc Electrophysiol.

[24] Thom T, Haase N, Rosamond W, Howard VJ, Rumsfeld J, Manolio T (2006). Heart disease and stroke statistics—2006 update: a report from the American Heart Association Statistics Committee and Stroke Statistics Subcommittee. Circulation.

[25] Benjamin IJ, Christians E (2002). Exercise, estrogen, and ischemic cardioprotection by heat shock protein 70. Circ Res.

[26] Minson CT, Halliwill JR, Young TM, Joyner MJ (2000). Influence of the menstrual cycle on sympathetic activity, baroreflex sensitivity, and vascular transduction in young women. Circulation.

[27] Saleh TM, Connell BJ (2003). Estrogen-induced autonomic effects are mediated by NMDA and GABAA receptors in the parabrachial nucleus. Brain Res.

[28] Mohamed MK, El-Mas MM, Abdel-Rahman AA (1999). Estrogen enhancement of baroreflex sensitivity is centrally mediated. Am J Physiol.

[29] Breuer HW, Skyschally A, Schulz R, Martin C, Wehr M, Heusch G (1993). Heart rate variability and circulating catecholamine concentrations during steady state exercise in healthy volunteers. Br Heart J.

[30] Carnethon MR, Jacobs DR, Sidney S, Sternfeld B, Gidding SS, Shoushtari C (2005). A longitudinal study of physical activity and heart rate recovery: CARDIA, 1987–1993. Med Sci Sports Exerc.

[31] Daugherty SL, Magid DJ, Kikla JR, Hokanson JE, Baxter J, Ross CA (2011). Gender differences in the prognostic value of exercise treadmill test characteristics. Am Heart J.

[32] Vivekananthan DP, Blackstone EH, Pothier CE, Lauer MS (2003). Heart rate recovery after exercise is a predictor of mortality, independent of the angiographic severity of coronary disease. J Am Coll Cardiol.

[33] Nishime EO, Cole CR, Blackstone EH, Pashkow FJ, Lauer MS (2000). Heart rate recovery and treadmill exercise score as predictors of mortality in patients referred for exercise ECG. JAMA.

[34] Pichon AP, de Bisschop C, Roulaud M, Denjean A, Papelier Y (2004). Spectral analysis of heart rate variability during exercise in trained subjects. Med Sci Sports Exerc.

[35] Kannel WB, Wilson PW, D'Agostino RB, Cobb J (1998). Sudden coronary death in women. Am Heart J.

[36] Benjamin EJ, Levy D, Vaziri SM, D'Agostino RB, Belanger AJ, Wolf PA (1994). Independent risk factors for atrial fibrillation in a population-based cohort. The Framingham Heart Study. JAMA.

[37] Dewland TA, Androne AS, Lee FA, Lampert RJ, Katz SD (2007). Effect of acetylcholinesterase inhibition with pyridostigmine on cardiac parasympathetic function in sedentary adults and trained athletes. Am J Physiol Heart Circ Physiol.

[38] De Meersman RE (1993). Heart rate variability and aerobic fitness. Am Heart J.

[39] Davy KP, Miniclier NL, Taylor JA, Stevenson ET, Seals DR (1996). Elevated heart rate variability in physically active postmenopausal women: a cardioprotective effect?. Am J Physiol.

[40] Byrne EA, Fleg JL, Vaitkevicius PV, Wright J, Porges SW (1996). Role of aerobic capacity and body mass index in the age-associated decline in heart rate variability. J Appl Physiol.

[41] Lazoglu AH, Glace B, Gleim GW, Coplan NL (1996). Exercise and heart rate variability. Am Heart J.

[42] Tulppo MP, Makikallio TH, Seppanen T, Laukkanen RT, Huikuri HV (1998). Vagal modulation of heart rate during exercise: effects of age and physical fitness. Am J Physiol.

[43] Maestri R, Pinna GD, Robbi E, Capomolla S, La Rovere MT (2005). Noninvasive measurement of blood pressure variability: accuracy of the Finometer monitor and comparison with the Finapres device. Physiol Meas.

[44] Dinenno FA, Jones PP, Seals DR, Tanaka H (2000). Age-associated arterial wall thickening is related to elevations in sympathetic activity in healthy humans. Am J Physiol Heart Circ Physiol.

[45] Schwartz PJ, La Rovere MT, Vanoli E (1992). Autonomic nervous system and sudden cardiac death. Experimental basis and clinical observations for post-myocardial infarction risk stratification. Circulation.

